# The need for ecological nested models as emerging theoretical frameworks in the investigation of affective variables in L2 education^☆^

**DOI:** 10.1016/j.heliyon.2023.e16891

**Published:** 2023-06-02

**Authors:** Xi Li

**Affiliations:** School of Foreign Languages, Henan University of Animal Husbandry and Economy, Zhengzhou, Henan, 450003, China

**Keywords:** **Key terms:** affective variables, Ecological study, SLA, Complex dynamic system theory, Dynamicity of emotions

## Abstract

As an emotionally dynamic process, language learning is marked by fluctuations in students' affective constructs (e.g., enjoyment, playfulness, boredom). Thus, clues supporting an ecological perspective on behavioral trends and changes in language learners' emotional constructs driven by relational person and contextual components may be discovered. Therefore, an ecological approach enriched with the nested ecosystems model and complex dynamic system theory (CDST) can facilitate the investigation of dynamics of language students' affective constructs. These are first categorized and analyzed on the microsystem scale concerning students' motivation, beliefs, linguistic factors, cognitive factors, affective factors, and classroom context. Then, they are analyzed in 3 ecosystems including meso-, exo-, and macrosystems, each revealing an invaluable dimension of the reality of the emergent dynamicity of the variables. The present paper first draws attention to the dynamic nature of L2 learners’ emotional factors and raises awareness of the ecological dynamic system theory. Then, it reviews the existing ecological studies of L2 affective variables and summarizes the findings. It ends with several conclusive remarks and suggestions for the future line of research.

## 1 Introduction

Individual affective variables play a significant role in the language learning process [1,2]. Indeed, language learning is viewed as a dynamic system including both affective and cognitive facets, with dynamic trajectories within and among individuals [[3], [4], [5], [6]]. Students may not pay attention to the impact of emotional aspects such as enjoyment, anxiety, and boredom on their language learning process. Nevertheless, they are continually impacted by the dynamic nature of these emotional factors [[6], [7], [8], [9], [10], [11], [12]]. L2 students' emotions, thus, have a great impact on the persons' current situations [[13], [14], [15]]. Positive emotions can help to stabilize intrapersonal and interpersonal abilities [[16], [17], [18], [19], [20], [21]], but negative emotions may have a debilitative impact on the engagement and performance for language learners [[22], [23], [24], [25], [26]]. In the learning process, both positive and negative emotions exhibit distinctive behavioral patterns that are mainly shaped in accordance with the power of internal or external variables. L2 students might start their language learning journey at a specific affective state [27]. However, under the impact of both internal and external variables, these initial situations can change [28]. For example, students' and instructor's responses and feedback from peers or teacher may cause them to exhibit different emergent emotional reactions.

It is also confirmed that even seemingly insignificant tiny shifts in learners' emotional states could still have a significant great effect on the entire network of class events [29,30]. Considering the different research on the affective factors of L2 students reveals that they primarily used a trait-oriented design to investigate these components. Surveys using questionnaires are unable to capture the sudden, unanticipated shifts in the students' affective characteristics [15,31]. Because of this, novel research techniques are required to investigate the dynamics of students' affective processes [6,27,32,33].

In addition, based on Larsen-Freeman's [34] argument, it might be assumed that the multi-level processes that underpin language acquisition are rarely linear and instead appear to emerge differently in various students at various stages or phases in time. As a result, shifting from a state-oriented perspective to one that is process-based and dynamic is necessary when examining the emotional variables of language learners [30]. The main objective of the current review is to address the significance of ecological nested models for the exploration of affective constructs in L2 education.

## 2 Complex dynamic systems theory

The advocators of complex dynamical systems theory (CDST) in the realm of SLA have been affected by social science fields that offer verification that most of contemporary critical challenges are complex and dynamic and must be treated in this manner [35] The first advocate for openly addressing SLA concerns from a complex dynamic approach was Diane Larsen-Freeman [36]. Language acquisition, according to CDST, is dynamic and develops or self-organizes into distinct patterns as a result of interactions between various context-bound elements. CDST employs an ecological perspective, originated in post-structuralism, in which the momentary and spatial setting as well as between and within individual differences have a critical effect on the trajectories of language-related factors [36]. In other words, students are not detached from their learning process but they are tied to their learning ecology. This is because of the fact that complex systems are self-organizing, co-adaptive, interconnected, and hence are regarded as transdisciplinary [36].

## 3 Ecological dynamic systems theory

A theoretical approach that could reflect the dynamicity of development in language learners’ affective variables is the ecological dynamic system theory, which is applied to investigate the network of relations between an organism (here an L2 learner) with the other organisms (instructor and classmates) engaging in a particular learning context characterized by emerging values, intrinsic dynamicity, tasks, diversity, and fluctuation [37]. An ecological view of a certain variable is related to the association of individuals and their surrounding world. Concerning this, the ecological approach clarifies the interplay between the L2 students and the verbal and nonverbal variables (e.g., cognitive, affective) embedded in the ecology of the language class [38,39]. As a result, ecological investigations of language students' affective factors illuminate the interactions between language students and their surrounding environment and may therefore provide fresh perspective into how affordances or agents trigger the growth or amplification of a specific affective component (e.g., boredom, enjoyment, playfulness, etc.). In fact, the ecological perspective holds an emic subjective or inner perspective towards the emergence of affective constructs in SLA. This means that the contributing ecosystemic factors underlying the construction of an affective factor such as enjoyment or anxiety are viewed from the perspective of the participants in a study not from an outside perspective. It additionally offers the benefit of taking into account the students' immediate context as an important factor influencing the development of various affective constructs [40]. As Larsen-Freeman [36] points out, it is impossible to adequately describe teaching or learning without referring to the context with which they are associated.

As for the classroom ecology, according to Larsen-Freeman [36], the parts that make up the learning system include not only the agents (i.e., teachers and students along with their perspectives, emotions, achievement, and behaviors), but also the physical and chronological features of the instructional surroundings. In fact, time spent in class, physical qualities, and anything related to time and space may have a substantial impact on teaching and learning. Hence, to investigate the emergence of any learning-related factor, researchers must evaluate it as it is embedded within all of the practicalities of the learning context. That explains why an ecological perspective is important in this case. It takes into account the primary contextual aspects, both human and nonhuman, that might all impact on the growth and fluctuations in the affective states of language learners' behavior. From an ecological viewpoint, language is seen as systematic and fraught with developmental patterns [41]. Thus, it seems interesting to uncover the unexpected patterns of emotions emerging from a nested network of ecosystems; yet, the existing body of research in SLA studies is still limited in this regard.

As noted by Van Lier [37], the apparently too much emphasis on emergence is due to the fact that language learning occurs when basic components are merged to form a broader system. According to Larsen-Freeman [36], emergence occurs when something new arises unexpectedly from a set of interrelated links among the constituent components (of a set). Therefore, examining students' emotional constructs from an ecological standpoint elucidates how diverse interplaying components at various situated states might contribute to the formation of a particular emotional state. When it comes to the diversity in the ecology of language acquisition, the consequence is that instructors should approach pupils individually and respect their differences [42,43].

Conclusively, an ecological perspective to investigating L2 students' affective factors provides a more comprehensive view of how a specific affective state (e.g., enjoyment, boredom, grit, etc.) might develop distinctively across different students. Implementing the steps of an ecological method and the assumed dynamic nature of affective constructs of language students [30,44], the justification for using nested ecosystems model together with the CDST is the primary focus of exploring the ecological components [37], the operative mechanisms, as well as the interactive components of the macro and micro systems [45]. In other words, the mentioned frameworks see ecology of language class [36] in a nonlinear, holistic, not reductionist, ever-changing, and emic way [37]. Despite many similarities between CDST and an ecological perspective, the ecological perspective sees the factors responsible for the emergence of an L2 affective construct from the insider perspective of the individuals and the concept of change is not a matter of debate in this perspective as it is in the CDST.

## 4 Related literature on ecological dynamic systems theory in assessing L2 affective variables

Under the influence of the dynamic phase in the realm of SLA [46], the L2 emotional factors such as enjoyment, boredom, and anxiety in recent years have been explored as situational states rather than traits with a subjective inner perspective. Regarding this inner perspective, Tran et al. [47] indicated that what language teachers regard as facilitative anxiety was not reciprocated by their learners. That is, language learners in this study did not believe in the facilitative aspect of anxiety but for all of them anxiety was associated with a negative experience. Also, the issue of ergodicty in the exploration of individual variations in the process of language learning implied that the average indicator of L2 affective variables does not necessarily reflect the individual indictors because language students are not ergodic ensembles [48]. These movements in the L2 affective domain have made this domain a suitable one for the exploration of its variables from an ecological approach with its focus on the subjective viewpoints of individual language learners regarding these variables in a given context. The difference between a contextual perspective and an ecological perspective lies in the fact that in a contextual perspective, different participants from different contextual layers are involved in the data collection. But from an ecological pint of view, the ecosystemic factors accounting for the emergence of an L2 affective variable are explored from the insider perspective of the individual participants in the study. Since the insider subjective perspective of individuals shapes the core of an ecological perspective, variability has a substantial impact on seeing how the underpinning themes of the emergence of an L2 affective factors are derived from an integration of divergent viewpoints. With respect to the subjectivity which is the focus of an ecological perspective, heterogeneity rather than homogeneity is emphasized in the selection of the participants. Thus, divergent sampling and extreme or deviant case sampling are prioritized in the sampling strategy of an ecological perspective. With respect to the role of emergence, context, divergence, and variability as the premises of an ecological perspective, any method which incorporates these premises can be regarded as a compatible method with an ecological perspective.

However, the ecological studies of language students' affective factors are still rare in SLA research [49]. The existing literature belongs to the past couple of years, yet the interesting findings point to the promising application of this method in investigating different dynamic aspects of the language learning experience. The main ecological models for the exploration of L2 affective factors are Bronfenbrenner’ nested ecosystems model [50] and activity theory [51]. A review of these studies is presented in Table 1. More elaborations will follow including more details on the underlying procedures of the ecological approach.

**Table 1:** A review of the ecological studies of L2 learners’ affective variables.

Title of paper	Authors (year)	Journal	Purpose of study	Main findings
Investigating situational willingness to communicate within second language classrooms from an ecological perspective	Cao (2011)	System	To explore the dynamic and situated quality of willingness to communicate in L2 classrooms	Situational willingness to communicate in EFL classes resulted from the interactive effects of individual factors, class contextual conditions and linguistic constructs.
Towards an ecological understanding of willingness to communicate in EFL classrooms in China	Peng (2012)	System	To explore factors affecting willingness to communicate in EFL classes in China	The effect of the meso-, exo-, and macrosystem on classroom willingness to communicate was proven.
On the exploration of the ecology of English language teachers' personal styles in Iran	Elahi Shirvan, Rahmani, & Sorayyaee (2016)	Asian-Pacific Journal of Second and Foreign Language Education	To investigate Iranian EFL teachers' personal styles from an ecological perspective	The teachers' personal style constructed in the class was dynamic and changed within the specific features of each class.
An ecological study of foreign language writing anxiety in English as a foreign language classroom	Saghafi, Adel & Zareian (2017)	Journal of Intercultural Communication Research	To use an ecological approach to learn more about L2 learners' anxiety in writing skill	Significant evidence was found to support the varying trajectories and factors affecting students' writing anxiety in a network of individual and environmental factors.
Iranian English language learners' attitude towards their accent in English language: An ecological approach	Rajablou & Elahi Shirvan (2017)	Language Teaching Research	To explore the attitudes of Iranian EFL learners' attitude to L1-accented English based on the nested ecosystems model	A prevalent emerging pattern of preference for native-like accent was found in the ecology of Iran marked by the cherishment of English with L1 accent.
Ecological understanding of foreign language speaking anxiety: Emerging patterns and dynamic systems	Kasbi & Elahi Shirvan (2017)	Asian-Pacific Journal of Second and Foreign Language Education	To explore EFL learners' speaking anxiety using nested ecosystems model and CDST	Evidence was found for an ecological understanding of patterns and variables affecting changes in students' speaking anxiety influenced by interconnected individual and environmental constructs.
The wellbeing of language teachers in the private sector: An ecological perspective	Mercer (2020)	Language Teaching Research	to investigate the wellbeing of English language teachers affiliated with the private sector	Evidence was found for how the teachers' wellbeing was determined by the business model feature in the private section in the form of working conditions and status of the ELT profession. Signs of positivity were revealed too.
The dynamics of foreign language enjoyment: An ecological momentary assessment	Elahi Shirvan, Taherian & Yazdanmehr (2020)	Frontiers in Psychology	To explore the different dynamic aspects of foreign language enjoyment across different timescales in an EFL course	Enjoyment in L2 fluctuated in the hierarchy of timescales, from momentary changes to the monthly changes.
Affordances in Iranian English for academic purposes education: An ecological approach	Fotuhabadi, Elahi Shirvan, & Khajavy (2020)	Human Arenas	To investigate ecological factors accounting for the actualized classroom affordances in EAP university classes using a nested ecosystem model	Possible affordances of EAP courses were primarily actualized in terms of affordance levels, at perception level, and no space was found for using the perceived affordances.
Affordances of the microsystem of the classroom for foreign language enjoyment	Elahi Shirvan & Taherian (2022)	Human Arenas	To investigate the potential affordances for foreign language enjoyment actualized in a listening and speaking university course using an ecological approach	The findings showed not all possible affordances for foreign language enjoyment are actualized as the applied affordances in the classroom context and only some can be used as the affordances shaped.

One of the first works of SLA research using the ecological approach was conducted by Cao [50] to explore language learners' willingness to communicate. This researcher firstly drew attention to the lack of attention to the dynamicity and situated nature of the variable in the pre-existing literature. Then, this study adopted an ecological perspective to L2 learning and conducted a multiple case study to explore the dynamic development of willingness to communicate in L2 classes. Several methods were used together including observations of classes, interviews in the form of stimulated-recall, as well as students' journals. The findings showed that, in L2 classes, situational willingness to communicate grew out of the joint effects of individual traits such as personality, self-confidence, emotions and perceived chances for communication, environmental conditions of classes including the tasks and activities, topics, the teacher, peers, and the size of group-based tasks, as well as the linguistic variables. Cao [52] concluded that L2 teachers need to be aware of the interconnected nature of all these affective, cognitive, and linguistic factors if they hope to increase L2 learners' willingness to communicate in class.

Language learners' willingness to communicate was also explored by Peng [53] in a multiple-case study. The researcher sought to find the network of factors affecting the target variable in the EFL setting of China. The participants were university students, who were interviewed and whose performance was also observed for seven months. Their journals were also used as another source of data to be analyzed. A qualitative data analysis followed. Bronfenbrenner's [54] nested ecosystems model was used as the model for analysis. The results showed that six factors accounted for the language learners' willingness to communicate in class as the microsystem. These included L2 learners' motivation, beliefs, linguistic, cognitive, affective factors, and classroom context. The findings also showed the presence of meso-, exo-, and macrosystems affecting willingness to communicate in class. These results provided empirical evidence for a better ecological knowledge of EFL learners' willingness to communicate in China. The researcher suggested that language learners' willingness to communicate was constructed socio-culturally out of a network of individual and contextual factors.

Additionally, ecological studies in SLA research addressed teachers. For instance, Elahi Shirvan, Rahmani and Sorayyayee [55] explored language teachers' personal styles. These researchers firstly emphasized that a linear approach should be avoided to the description of a complex dynamic variable in SLA research. They emphasized that more ecological investigations were required on L2 teacher-related variables. Therefore, in their research, they investigated Iranian EFL teachers' personal styles using an ecological perspective. The styles they explored included controlling vs nurturing parent, adult, adopted vs. natural child. Semi-structured interviews, journal writing, and observations were used to collect the data. A qualitative analysis followed using the nested ecosystems model of analysis. Finally, the internal and external dimensions underlying the instructors' personal styles were identified at the micro system. Also, the data analysis proved the presence of meso, exo, and macrosystems and their influence on the instructors' personal styles. This study showed that the construction of teachers' personal styles was very dynamic in class. Personal styles changed within the specificities of each class concerning the variables addressed in this research. The researchers finally further discussed the systematic intervention of every construct related to the teachers’ specific styles.

In a more recent work, Saghafi, Adel, and Zareian [56] explored the anxiety language learners experienced in writing, as an affective reaction to the dynamicity involved in the SLA procedure which made learners make great changes to their emotional states. Saghafi et al. [56] adopted an ecological approach in the nested ecosystem model in their study. Their stud was carried out on a number of upper-intermediate EFL learners, between 14 and 18 years of age. The data collection procedures included class observations, semi-structured stimulated recall, journal writing, and task-motometer for a whole 10 sessions of classroom learning to give information about learners' anxiety experienced during writing that was qualitatively analyzed. According to the principles of nested ecosystem model, the emergent patterns of learners' anxiety expressed in writing were categorized in micro-, meso-, exo-, and macrosystems. Saghafi et al. [56] provided enough evidence that supported the changing trajectories and variables related to language learners’ anxiety during the writing tasks embedded in the interconnection of the individual and contextual factors.

Moreover, Anxiety was ecologically explored in the speaking skill by Kasbi and Elahi Shirvan [57]. A similar procedure was followed by these researchers to pursue speaking anxiety dynamically in the Iranian context. A nested ecosystems model along with the complex dynamic system theory were employed. Semi-structured interviews were conducted for five sessions, non-participant observation in the class and anxiometers were used for data collection. They helped to unravel the dynamics of language learners' anxiety within 5 sessions. A qualitative analysis followed next. Finally, the researchers categorized the emergent patterns of learners' speaking anxiety initially at the microsystem level regarding language learners’ motivation, beliefs, linguistic factors, cognitive variables, affective constructs, and classroom setting. The researchers finally discussed the students' anxiety in 3 ecosystems comprised of meso-, exo-, and macrosystems. The students' anxiety was also explored according to the dynamic behaviours of change and stability in the students' micro progress. This research provided proof for an ecological interpretation of the variables and patterns included in language students' varied speaking anxiety effected by the interactive individual and environmental variables.

In the same year, Rajablou and Elahi Shirvan [58] adopted the same nested ecological framework to explore language learners' attitudes to their accented English speaking skill in the EFL context. They used the nested ecosystems model of Bronfenbrenner [54] comprised of micro-, meso-, exo-, and macro-systems. They also employed triangulated data collection using a questionnaire to explore learners' attitudes completed by 157 respondents (of both sexes) as well as semi-structured interviews. Within the ecology of Iran, these researchers found the prevalent emerging pattern of preferring native-like accent while acknowledging English with a Persian accent. The factors that influenced to the emergent behavior of preferring native-like accent as embedded in the Iranian setting were: linguistic security maintenance and self-confidence, teacher role, materials included in the class microsystem, learners’ prior experiences in the mesosystem, English language institute policies at the exosystem, and overall attitude about accent in the macrosystem.

Furthermore, in 2020, Mercer [59] investigated EFL teachers' well-being in the private sector of Malta. For data collection, semi-structured interviews along with visual cues and journal writings were used. A grounded interpretative phenomenological analysis followed. The ecological perspective was applied to the evaluation of data. The major findings indicated how the instructors’ well-being was influenced by the business model properties of the private sector, especially concerning the work conditions and the prestige of the language teaching profession in the community of interest. It most often means precarity for the language teachers, concerning the job and prospective success. Yet, the study also identified signs of positivity. For instance, instructors were revealed to enjoy their instructions, constructive affairs with co-workers and learners, and a positive work atmosphere, that changed from one institution to another. The findings of this research had implications for effective practice in the SLA field.

The line of ecological studies in SLA domain in 2020 continued with the study conducted by Elahi Shirvan, Taherian and Yazdanmehr [4] influenced by the recent shift from negative psychology to positive psychology. Elahi Shirvan et al. [4] explored the trajectories of language students' foreign language enjoyment in the field of SLA. These researchers used an ecological momentary assessment in order to complement the current understanding of the person-centered dynamics of this ecosystem in the context of language learning classroom. They applied a time-based sampling scheme of ecological momentary assessment and explored the dynamic nature of enjoyment within various time points including seconds, and minutes in an intermediate language course. The researchers utilized open-ended interviews as well as recorded journals for weeks. Though their research did not follow a nested ecological approach, the findings were useful as they adopted a chronosystemic approach and indicated fluctuation in the sequence of time scales, from moment-to-moment fluctuations to the monthly fluctuations. It is worth noting that among the main ecosystems which are the focus of ecological studies, chronosystem refers to the timing in the emergence of psychological constructs such as L2 affective variables. They argued the behavioral trend of enjoyment from one time-frame to another based on the characteristics of the CDST.

In another ecological study, Fotuhabadi, Elahi Shirvan, and Khajavi [60] investigated the ecological factors affecting the realization of class affordances in a number of English for academic courses in Iran. They employed a nested ecosystem model as for analysis. They employed a modern social science hermeneutics approach and interviewed some English for academic purposes university lecturers and a few students in these courses. Class observations followed too. In terms of affordance levels, the results showed that the possible affordances of the target classes were primarily realized on a perception scale. No space was observed for the realization of the affordances perceived. However, the lecturers were not agent in constructing the affordances shaped in these courses. In addition, the teachers were not provided with any formal EAP training course so as to become acquainted with the acquired research results. Neither have they provide support for more cooperation of instructors with the English language teachers. Fotuhabadi et al. (2020) finally discussed the latent causes of these problems within micro-, meso-, exo-, as well as macrosystems.

The latest work of ecological research in SLA domain was the study by Elahi Shirvan and Taherian [61] which revealed how the potential affordances for foreign language enjoyment were actualized in a university listening and speaking course from an ecological perspective. The ecological framework helped the researchers to reveal a realistic image of the contextual process of actualizing foreign language enjoyment affordances within the microsystem of the classroom. Two rounds of interview were used with the teacher of the course. This analysis revealed that not all the potential affordances for foreign language enjoyment were realized as the affordances used in the classroom context and just a few could translate into the shaped affordances. These results showed that limiting the actual affordances for foreign language enjoyment to perceived affordances or extending them to the affordances used in an EFL class is a function of the match or mismatch between the structure of class in the microsystem and the rules and regulations of the school as the exosystem.

## 5 Conclusion

The review of the above-mentioned works of research highlights the importance of the ecological perspective to examining diverse emotional factors embedded in learning a language. Bronfenbrenner's [54] analytical framework of nested ecosystems model, as used and found in the above-mentioned studies, recurrently highlights the social embeddedness of learner (or teacher) led affective variables. The findings of the existing literature addressed the socio-cultural features of various emotional constructs such as enjoyment and boredom within the interplay systems of external and internal factors. In this regard, tracing the behavioral trend of the dynamic nature of language learners' emotional states with respect to beliefs, consistency, fluctuation, and action within the class ecology enables researchers to identify relevant consequences on a multi-systemic scale. The existing literature showed that several factors were involved including the students' motivation, attitudes and, linguistic and cognitive resources in impacting on the growth of the learners' emotional factors at the micro-system. Then, at the mesosystem level, the effect of students' previous learning experience and participation in extracurricular activities was prominent. Next, at the exo-system level, components such as classroom context, the curriculum and, assessment system were influential. Finally, at the macro-system level, the important impact of social, cultural and educational elements was highlighted.

Moreover, the findings of the above-mentioned literature proved the essentiality of acknowledging the ecological nature of the SLA-related topics. The student or teacher-related affective factors indicated that they emerged dynamically from an associative nested system of cognitive, linguistic, and affective elements. It is also possible to argue that the contextual aspects of language teaching or learning systems cannot be completely predictive of upcoming events. Therefore, the generalizability of the results to other learners in different settings should be limited [62]. As described by Pennington and Hoekje [63], language learning and teaching takes place as an interaction of different levels, probably as a disciplinary field, a profession, a significant life-changing domain, and a service. They report that, the ELT domain is beyond the individuals and individual programs. Therefore, the English language learning or teaching experience should be seen not as a unitary task but rather as an ecosystem [63]. Thus, the whole language teaching and learning community such as the school principals, teachers, students, curriculum developers and academics should get somehow involved in the network of working and learning conditions from context to context, the status of the teacher or learner, concept of the professional teacher or learner [64], and the main relevant topics including academic justice and native speakerism. Positive changes will happen only when all those involved, at the different levels of ecologies, cooperate hand in hand to improve the quality of the lives of language teachers and learners.

## 6 Suggestions for further research

Following the enlightening discoveries of ecological research in the SLA domain, it is expected that this avenue of study can further provide insights into other emotional factors that have not yet been investigated ecologically in language research. Such variables as willingness to communicate, anxiety and foreign language enjoyment are explored more than others, while some are significantly left unexplored at least from an ecological perspective. These can include foreign language students' boredom [10], playful learning, L2grit and so on. More empirical evidence is needed about how these affective variables dynamically emerge from the real classroom learning experience. Multiple ways of collecting data together such as stimulated recalls, interviews, class observations and journal writing (as the existing literature employed) and a nested ecological analytical model can reveal much about the external and internal factors affecting the growing patterns of these variables. Due to the inner subjective orientation of an ecological perspective, some researchers might face challenges in the data collection and the interpretation of the findings as they need to be done very meticulously and systematically. Thus, it is highly recommended that researchers interested in an ecological perspective to L2 affective variables should improve their literacy of qualitative data analysis and interpretation, and the well-established evaluative criteria for the evaluation of qualitative research [65].

As investigated in a few studies reviewed above, the affective variables such as anxiety can be explored as involved in different language skills. Accordingly, two of the works cited above explored this affective construct in language learners' writing and speaking. Further research can be done to explore it in the other two language skills as well and compare the results. The results of the multiple time-scaled research above indicated nuance perspective on the fluctuations of the target factors. Additional research is required to be able to closely and realistically monitor developments in the constructs of interest. In spite of the increasing awareness of positive psychology, it is proposed that, due to a lack of ecological studies in SLA, both positive and negative emotional factors (which play a key role in language acquisition) should be investigated via the employment of a nested framework of ecological systems. The ecological perspective intertwined with the dynamic shift in the L2 affective domain can direct the research orientation toward these variables to a dynamic-ecological phase of research in which deeper insights into the variables of this domain can be achieved via an ecologically subjective perspective.

## Author contribution statement

All authors listed have significantly contributed to the development and the writing of this article.

## Data availability statement

No data was used for the research described in the article.

## Additional information

No additional information is available for this paper.

## Declaration of competing interest

The authors declare that they have no known competing financial interests or personal relationships that could have appeared to influence the work reported in this paper.
